# Ubiquitination of NKCC2 by the cullin-RING E3 ubiquitin ligase family in the thick ascending limb of the loop of Henle

**DOI:** 10.1152/ajprenal.00079.2022

**Published:** 2023-02-01

**Authors:** Gustavo R. Ares

**Affiliations:** ^1^Hypertension and Vascular Research Division, Department of Internal Medicine, Henry Ford Hospital, Detroit, Michigan, United States; ^2^Department of Physiology, Integrative Bioscience Center, Wayne State University, Detroit, Michigan, United States

**Keywords:** cullin-RING E3 ubiquitin ligase complex, E3 ligase, neddylation, renal transporter

## Abstract

The Na^+^/K^+^/2Cl^−^ cotransporter (NKCC2) in the thick ascending limb of the loop of Henle (TAL) mediates NaCl reabsorption. cGMP, the second messenger of nitric oxide and atrial natriuretic peptide, inhibits NKCC2 activity by stimulating NKCC2 ubiquitination and decreasing surface NKCC2 levels. Among the E3 ubiquitin ligase families, the cullin-RING E3 ubiquitin ligase (CRL) family is the largest. Cullins are molecular scaffold proteins that recruit multiple subunits to form the CRL complex. We hypothesized that a CRL complex mediates the cGMP-dependent increase in NKCC2 ubiquitination in TALs. Cullin-1, cullin-2, cullin-3, cullin-4A, and cullin-5 were expressed at the protein level, whereas the other members of the cullin family were expressed at the mRNA level, in rat TALs. CRL complex activity is regulated by neuronal precursor cell-expressed developmentally downregulated protein 8 (Nedd8) to cullins, a process called neddylation. Inhibition of cullin neddylation blunted the cGMP-dependent increase in ubiquitinated NKCC2 while increasing the expression of cullin-1 by threefold, but this effect was not seen with other cullins. CRL complex activity is also regulated by cullin-associated Nedd8-dissociated 1 (CAND1). CAND1 binds to cullins and promotes the exchange of substrate-recognition proteins to target different proteins for ubiquitination. CAND1 inhibition exacerbated the cGMP-dependent increase in NKCC2 ubiquitination and decreased surface NKCC2 expression. Finally, cGMP increased neddylation of cullins. We conclude that the cGMP-dependent increase in NKCC2 ubiquitination is mediated by a CRL complex. To the best of our knowledge, this is the first evidence that a CRL complex mediates NKCC2 ubiquitination in native TALs.

**NEW & NOTEWORTHY** The Na^+^/K^+^/2Cl^−^ cotransporter (NKCC2) reabsorbs NaCl by the thick ascending limb. Nitric oxide and atrial natriuretic peptide decrease NaCl reabsorption in thick ascending limbs by increasing the second messenger cGMP. The present findings indicate that cGMP increases NKCC2 ubiquitination via a cullin-RING ligase complex and regulates in part surface NKCC2 levels. Identifying the E3 ubiquitin ligases that regulate NKCC2 expression and activity may provide new targets for the development of specific loop diuretics.

## Graphical abstract

**Figure:**
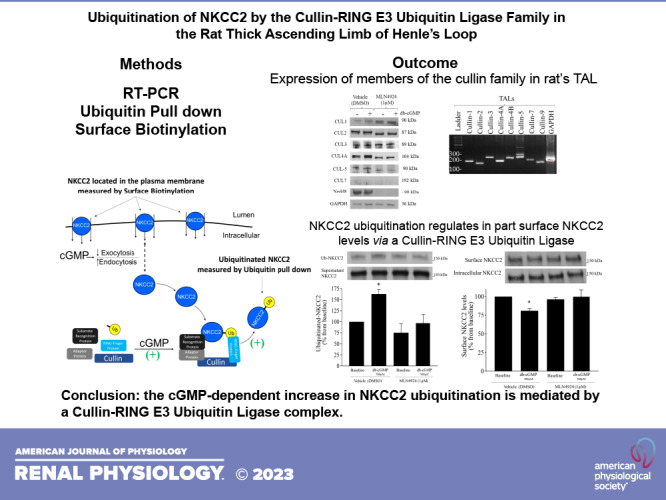

## INTRODUCTION

The kidneys play a major role in maintaining normal blood pressure by regulating NaCl reabsorption (1). In the kidney, the thick ascending limb of the loop of Henle (TAL) reabsorbs ∼30% of NaCl filtered through the glomeruli via the Na^+^/K^+^/2Cl^−^ cotransporter (NKCC2) (2, 3). NKCC2, located in the apical plasma membrane, mediates TAL-dependent NaCl reabsorption. We have shown that ∼5% of the total NKCC2 pool is located in the apical plasma membrane (4–6) maintained by a dynamic balance between endocytosis, exocytosis, recycling, and degradation by the proteasome (4–7).

cGMP, the second messenger of nitric oxide and atrial natriuretic peptide, exerts potent diuretic and natriuretic effects along the nephron (8–12). In the TAL, cGMP inhibits NKCC2 activity by decreasing surface NKCC2 levels (4). Moreover, we have recently shown that NKCC2 is ubiquitinated and that cGMP increases the rate of NKCC2 ubiquitination and degradation (7). However, the E3 ubiquitin ligases that mediate baseline and cGMP-stimulated NKCC2 ubiquitination are unknown.

Phosphorylation and glycosylation are posttranslational mechanisms known to participate in the regulation of surface NKCC2 expression and therefore NKCC2 activity (4–6, 13, 14). Recently, ubiquitination was added to the list of posttranslational modifications that regulate NKCC2 in the TAL (7, 15–17). Ubiquitination is a posttranslational modification that involves the participation of three enzymes [activating (E1), conjugating (E2), and ligase (E3) enzymes], which attach ubiquitin (a 76-amino acid peptide) to a target protein (18, 19). In humans, >600 E3 ubiquitin ligases have been categorized into four families (20–23). Alterations in the function or expression of E3 ubiquitin ligases have been linked to human disease (20, 24–33). Among the four main families of E3 ubiquitin ligases, the largest family is the cullin (CUL)-RING E3 ubiquitin ligase (CRL) complex, which mediates the ubiquitination of ∼20% of cellular proteins degraded by the proteasome (34). Cullins are the central component of the CRL complex and act as scaffold proteins, bringing together several multisubunit proteins including the RING-finger protein, adaptor proteins, and substrate-recognition proteins to form an active complex that targets proteins for ubiquitination (35).

The CRL complex is dynamically regulated by the conjugation and deconjugation of neuronal precursor cell-expressed developmentally downregulated protein 8 (Nedd8) with members of the cullin family (36–38). The conjugation of Nedd8 to cullin, called neddylation, activates the CRL complex (39, 40). Nedd8 is an 81-amino acid peptide with a molecular weight of 9 kDa. Nedd8 is considered a ubiquitin-like protein since it shares 60% identity and 80% homology with ubiquitin and is covalently conjugated to a limited number of proteins (41). Members of the cullin family differ within their NH_2_-terminal regions, whereas in the COOH-terminus, all members of the cullin family contain a lysine residue that is targeted by Nedd8 (20, 35). Cullin neddylation can be inhibited pharmacologically with MLN4924 (42), which is a potent and highly selective small-molecule inhibitor of the Nedd8 activating enzyme (NAE). By covalently binding to NAE to create a covalent Nedd8-MLN4924 adduct, MLN4924 blocks the first step of the cullin neddylation cascade (43), leading to the accumulation and inactivation of CRL complexes (32, 44). Therefore, by inhibiting cullin neddylation, we explored whether the ubiquitination of NKCC2 is mediated by a CRL complex.

On the other hand, CRL complex activity is also regulated by cullin-associated Nedd8-dissociated 1 (CAND1). CAND1 binds unneddylated cullin and promotes the exchange of substrate-recognition proteins to target different proteins for ubiquitination (45–48). CAND1 was originally found to interact with cullin-1 (49, 50). Later studies have shown that CAND1 also interacts with other members of the cullin family (45, 47). CAND1 binds members of the cullin family, promoting the exchange of substrate-recognition proteins and preventing cullin neddylation (51, 52). Therefore, by inhibiting CAND1 (53), we explored whether cGMP exacerbates the ubiquitination of NKCC2.

Dysfunction of the ubiquitin-proteasomal system has been linked to hypertension (15, 26–31, 33, 35, 54–60). This is the case in Liddle syndrome, in which a gain-of-function mutation in the epithelial Na^+^ channel (ENaC) prevents ENaC degradation (55, 61). Thus, ENaC remains constitutively active, causing increased Na^+^ reabsorption, which leads to hypertension. Thus far, the E3 ubiquitin ligase Nedd4-2 has been linked to Liddle syndrome (30). Another example is Gordon syndrome (29, 62) (also known as pseudohypoaldosteronism type II or familial hyperkalemic hypertension syndrome) (27, 31), in which a loss-of-function mutation in cullin-3 affects proteasomal degradation of the with-no-lysine (WNK) serine-threonine kinase family (WNK1 and WNK4) (28, 29, 58). A loss-of-function mutation in cullin-3 genes leads to an increase in Na^+^-Cl^−^ cotransporter (NCC) phosphorylation, leading to exacerbated NCC-dependent NaCl reabsorption in the distal nephron (62). Thus far, the E3 ubiquitin ligase cullin-3 has been linked to Gordon syndrome (27–29, 31).

Hypertension is the leading cause of “loss of health” worldwide. Approximately 30% of the United States population over 50 yr of age is hypertensive. A sustained increase in blood pressure caused by acute NaCl intake is defined as salt sensitivity. Salt sensitivity is found in 50% of African Americans and in 30% of Caucasians. Enhanced salt retention by the TAL has been described in patients with salt-sensitive hypertension (63, 64) and genetic animal models (65–70) of salt-sensitive hypertension. Moreover, higher NKCC2-mediated NaCl reabsorption has been linked to hypertension in animal models of salt-sensitive hypertension and in humans (63, 66–68, 71–74). However, the actual location and molecular mechanism by which NKCC2 participates in the regulation of blood pressure are unknown.

The E3 ubiquitin ligases involved in NKCC2 ubiquitination have not yet been identified. Therefore, we hypothesized that a CRL complex mediates the cGMP-dependent increase in NKCC2 ubiquitination in TALs. Since surface NKCC2 level is regulated by ubiquitination (7), understanding the mechanism by which NKCC2 is regulated by an E3 ubiquitin ligase may contribute to the development of new loop diuretics.

## MATERIALS AND METHODS

### Reagents

The composition of the physiological solution (PS) was as follows: 130 mM NaCl, 2.5 mM NaH_2_PO_4_, 4.0 mM KCl, 1.2 mM MgSO_4_, 6 mM l-alanine, 1.0 mM sodium citrate, 5.5 mM glucose, 2.0 mM calcium lactate, and 10 mM HEPES (pH 7.40). The reagents and antibodies used are shown in Table 1.

**Table 1.** Reagents and antibodies used in the present study

	Source	Cat. No.	Species	Dilution Used
*Antibody*				
NKCC2 (4–7)	In House		Rabbit	1/10,000
GAPDH	Chemicon	MAB374	Mouse	1/10,000
Cullin-1	Abcam	Ab75817	Rabbit	1/1,000
Cullin-2	Invitrogen	51-1800	Rabbit	1/1,000
Cullin-3	Sigma	SAB-4200180	Mouse	1/1,000
Cullin-4A	Invitrogen	PA5-14542	Rabbit	1/1,000
Cullin-4B	Invitrogen	PA5-51084	Rabbit	1/1,000
Cullin-5	Invitrogen	PA5-79100	Rabbit	1/1,000
Cullin-7	ProteinTech	13778-1-AP	Rabbit	1/1,000
Nedd8	Cell Signaling	2745S	Rabbit	1/1,000
CAND1	Abcam	ab183748	Rabbit	1/10,000
*Drug*				
db-cGMP	Biolog/Axxora	D010-50		100, 500, and 1,000 µM
MG132 (proteasome inhibitor)	Calbiochem	474790		20 µM
MLN4924 (NAE inhibitor)	Millipore	505477		1 µM
C60 (CAND1 inhibitor)	ChemDiv	E864-0360		1 µM

### Suspension of Medullary TALs From Male Sprague–Dawley Rats

Rat medullary TAL suspensions were obtained as previously described (4, 7). Male Sprague–Dawley rats weighing 200–250 g (Charles River Breeding Laboratories, Wilmington, MA) were fed a diet containing 0.22% sodium and 1.1% potassium (Purina, Richmond, IN) with water provided ad libitum for at least 7 days. On the day of the experiment, rats were anesthetized with ketamine and xylazine (100 mg/kg and 20 mg/kg body wt delivered intraperitoneally, respectively). The abdominal cavity was opened, and the kidneys were perfused retrogradely via the aorta with PS containing 0.1% collagenase (Sigma, St. Louis, MO) and 100 U/mL heparin. The inner strip of the outer medulla was cut into coronal slices, minced, and incubated at 37°C for 30 min with 0.1% collagenase in PS with gassing every 5 min with 100% oxygen. The tissue was pelleted by gentle centrifugation at 120 *g* for 2 min, suspended in chilled PS, and stirred on ice for 30 min to detach the tubules from each other. The suspension was filtered through 250-μm nylon mesh and centrifuged at 120 *g* for 2 min. The pellet was washed, centrifuged again, and finally suspended in 0.4 mL of chilled PS. In general, TAL suspensions were split into four aliquots for ubiquitin pulldown assays or experiments to study the biotinylation of plasma membrane proteins. All animal protocols were approved by the Institutional Animal Care and Use Committee of Henry Ford Hospital and Wayne State University.

### Pull Down of Ubiquitinated NKCC2 From Sprague–Dawley Rats

Ubiquitinated NKCC2 was measured as previously described (7). Briefly, ubiquitinated proteins were isolated using a Ubiqapture-Q kit (Enzo Life Sciences, Farmingdale, NY) following the manufacturer’s instructions. To measure ubiquitinated NKCC2 and the effect of cGMP, the whole TAL sample was treated with the proteasomal inhibitor MG132 (20 µM). TALs were split and treated with vehicle or inhibitor for 10 min at 37°C. Vehicle or dybutyryl cGMP (db-cGMP) was then added to the respective samples and incubated for 50 min at 37°C. Once the treatment was finalized, samples were cooled with chilled PS. Suspensions were centrifuged at 120 *g* for 2 min at 4°C. The PS was discarded, and TALs were incubated with lysis buffer [containing 150 mM NaCl, 50 mM HEPES, 5 mM EDTA, 2% Triton X-100, and 0.2% SDS and supplemented with protease inhibitors, namely, 10 µg/mL aprotinin, 5 µg/mL leupeptin, 4 mmol/L benzamidine, 5 µg/mL chymostatin, and 5 µg/mL pepstatin-A; pH 7.5 (Sigma)]. TALs were vigorously vortex three times for 3 s each. Each tube was spun at 12,000 *g* for 2 min at 4°C. The undissolved pellet was discarded. The protein content in each sample was measured in duplicate with a colorimetric assay using Bradford’s method (Pierce Biotechnology, Rockford, IL). The TAL lysate (150 μg protein) was incubated on a rocking platform at 4°C overnight with 40 μL of a 50% slurry containing Ubiqapture-Q beads in a final volume of 400 μL. The beads were centrifuged at 12,000 *g* for 2 min at 4°C. The supernatant was separated from the beads and saved for later measurement of nonubiquitinated NKCC2 and was also used as a loading control. The beads were washed twice with high-salt buffer (500 mM NaCl and 50 mM HEPES; pH 7.4) and twice with no-salt buffer (50 mM HEPES; pH 7.4). Proteins were eluted from the beads by boiling in 60 μL of SDS Laemmli loading buffer containing 50 μM *dl*-dithiothreitol and 5% β-mercaptoethanol. Proteins from the supernatant and proteins eluted from the beads were separated by SDS-PAGE (6% gels) and transferred to Immobilon PVDF membranes (Millipore, Bedford, MA). NKCC2 was detected by Western blot analysis.

### Surface Biotinylation of TAL Suspensions

Cell surface biotinylation of TAL suspensions was performed as previously described (4–7). Briefly, the TAL suspension was divided into four aliquots of equal volume. TALs were equilibrated for 10 min at 37°C and gassed every 5 min with 100% oxygen. After equilibration, TALs were treated with either vehicle or inhibitor for 20 min at 37°C with gassing every 5 min. After treatment, suspension samples were rapidly cooled to 4°C, washed twice with chilled PS, and centrifuged at 120 *g* for 2 min at 4°C. TAL samples were then incubated with 0.75 mL of chilled biotinylation solution (HEPES-Ca^2+^/Mg^2+^ buffer consisting of 10 mM HEPES, 130 mM NaCl, 2 mM MgSO_4_, 1 mM CaCl2, and 5.5 mM glucose; pH 7.8) containing 1.2 mg/mL NHS-SS-biotin (Pierce Biotechnology) on a rocking platform at 4°C for 15 min. Then, 0.75 mL of freshly prepared NHS-SS-biotin (1.2 mg/mL) was added to the top of the solution, and samples were incubated for an additional 15 min. After biotinylation, tubules were washed three times at 4°C with PS containing 100 mM glycine to remove excess NHS-SS-biotin. TALs were then centrifuged at 120 *g* for 2 min at 4°C and lysed in buffer (composition listed in a previous protocol). The protein content in each sample was measured in triplicate by colorimetric assay following the Bradford method (Pierce Biotechnology). Equal amounts of protein (50–75 μg) were incubated overnight at 4°C with streptavidin-coated agarose beads (10%) in lysis buffer. The beads were pulled down by centrifugation, and the supernatant was incubated with streptavidin-coated agarose beads (10%) for 2 h at 4°C. The supernatant was saved for use in determining intracellular NKCC2 levels, and the beads were centrifuged and pooled with the beads obtained from the first preparation. All beads were then washed twice in lysis buffer, twice in high-salt buffer (500 mM NaCl and 50 mM HEPES; pH 7.4), and twice in no-salt buffer (50 mM HEPES; pH 7.4). Biotinylated proteins were extracted from the beads by boiling in 60 μL of SDS-loading buffer containing 50 μM *dl*-dithiothreitol and 5% β-mercaptoethanol. The proteins were separated by SDS-PAGE (6% gels), and NKCC2 in the membrane was detected by Western blot analysis.

### Western Blot Analysis

Proteins eluted from streptavidin beads or obtained from TAL lysates were centrifuged for 1 min at 10,000 *g*, loaded into each lane of a 6% SDS-polyacrylamide (PAGE) gel, separated by electrophoresis, and transferred to Immobilon-P PVDF membranes. Membranes were blocked with 50 mM Tris, 150 mM NaCl, 5% nonfat dried milk, and 0.1% Tween 20 at pH 7.4 for 60 min and then incubated with primary antibodies (dilutions shown in Table 1) in 1% BSA as blocking buffer for 120 min at room temperature. The primary antibodies used to detect NKCC2, GAPDH, CUL1, CUL2, CUL3, CUL4A, CUL4B, CUL5, CUL7, Nedd8, and CAND1 are shown in Table 1. Horseradish peroxidase-labeled secondary antibodies were detected by chemiluminescence and quantified by densitometry. The membrane was washed twice for 15 min in buffer containing 50 mM Tris, 150 mM NaCl, and 0.1% Tween 20 at pH 7.4 and incubated with secondary antibody at a 1/1,000 dilution against the appropriate IgG conjugated to horseradish peroxidase (GE Healthcare, Chicago, IL). The chemiluminescence reaction was captured (GE Healthcare) on Kodak Biomax light film and quantified by densitometry. The film was scanned at a resolution of 1,200 dpi with 16-bit grayscale with an Epson 1680 Expression Pro scanner in positive film mode and saved as an uncompressed TIFF file. Software in transmittance mode was used to obtain the mean optical densities of the bands. The exposure time and amount of loaded protein were optimized such that the optical densities on the film were within a linear range.

### Reverse Transcriptase PCR Analysis

Isolated medullary TALs were obtained as previously described (75). Briefly, after anesthesia, the rat abdominal cavity was opened, and the left kidney was bathed in ice-cold saline and removed. Coronal slices were placed in oxygenated physiological saline. Medullary TALs were dissected under a stereomicroscope at 4°C−10°C. TALs were transferred to an Eppendorf tube. Total RNA was isolated from medullary TALs using TRIzol according to the instructions provided by the manufacturer (Life Technology, Invitrogen, Waltham, MA). RNA to cDNA conversion was performed using ∼1 μg of total RNA per sample using the One-Step iScript kit (Bio-Rad, Hercules, CA) in a reaction volume of 20 μL containing the reverse transcriptase enzyme. The resulting cDNA was diluted 1/10, and PCR was performed using Phusion Plus DNA Polymerase (1 μL), dNTPs (each at 0.2 mM), and magnesium sulfate (1.2 mM) as recommended by the manufacturer (ThermoFisher Scientific, Waltham, MA). The primers used for PCR analysis for each member of the cullin family were designed using the cDNA sequence with the intention to avoid introns or noncoding sequences. The primers were designed using Primers3Plus software (Boston, MA) and are shown in Table 2.

**Table 2.** Primers used in the present study

Protein	Primer		
	Forward (5′–3′)	Reverse (3′–5′)	Product Size, bp
Cullin-1	CACCTCCACACCAGAGGTTT	TGGAGACGCACATCTGAAAG	174
Cullin-2	TTTTCCTGGCAGCTAGCATT	ATCGGAACTGACACCACACA	154
Cullin-3	GTGTTTCCCTTGCTCCACAT	ACGGCAACAAGCCAAATAAC	227
Cullin-4A	TTGCTGCTTTGTCCCTCTTT	GTGTGAAAGCCAAGCTCCTC	180
Cullin-4B	CTGAGCTCTAATGCCCAAGG	TCTCTGGAACAGGAGGCACT	218
Cullin-5	GTGAGCCTTTGAGCATGTGA	CACAGGTGGGACTCCACTTT	233
Cullin-7	TCATTGTCTGAGCGTCCTTG	TTACAGATGGTTGCGAGCTG	194
Cullin-9	CAGATGAGGGGATGGAGAAA	TCACACCACTGTGACGGATT	165
GAPDH	AGACAGCCGCATCTTCTTGT	CTTGCCGTGGGTAGAGTCAT	207

### Statistical Analysis

**Figure 1.**
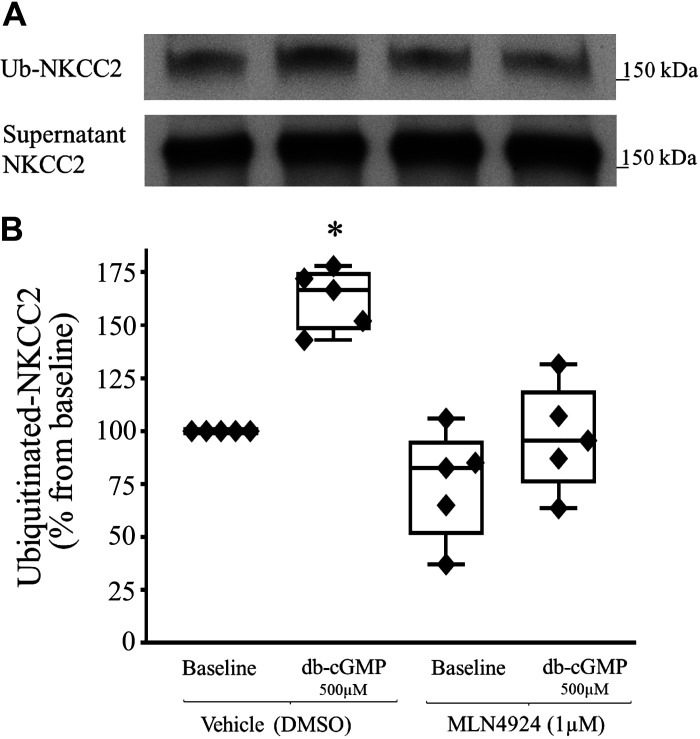
Effect of pharmacological inhibition of cullin activity on Na^+^/K^+^/2Cl^−^ cotransporter (NKCC2) ubiquitination. Inhibition of cullin neddylation with MLN4924 blunted the cGMP-dependent increase in NKCC2 ubiquitination. *A*: representative Western blots showing the effect of dibutyryl cGMP (db-cGMP) on NKCC2 ubiquitination. The physiological solution contained a proteasomal inhibitor (20 µM MG132) to stop the degradation of ubiquitinated NKCC2. A thick ascending limb of the loop of Henle (TAL) suspension was equilibrated at 37°C for 10 min with vehicle or neuronal precursor cell-expressed developmentally downregulated protein 8 activating enzyme (NAE) inhibitor (MLN4924, 1 µM), followed by 50 min of treatment with db-cGMP (500 µM). Intracellular NKCC2 expression did not change with treatment (*bottom*). *B*: cumulative data showing the effect of db-cGMP (500 µM) on ubiquitinated NKCC2 in the absence or presence of NAE inhibitor (MLN4924, 1 µM; *n* = 5, *P < 0.05 vs. basal). The data (means ± SE) are expressed as the percentage from baseline and were statistically analyzed by one-way ANOVA.

**Figure 2.**
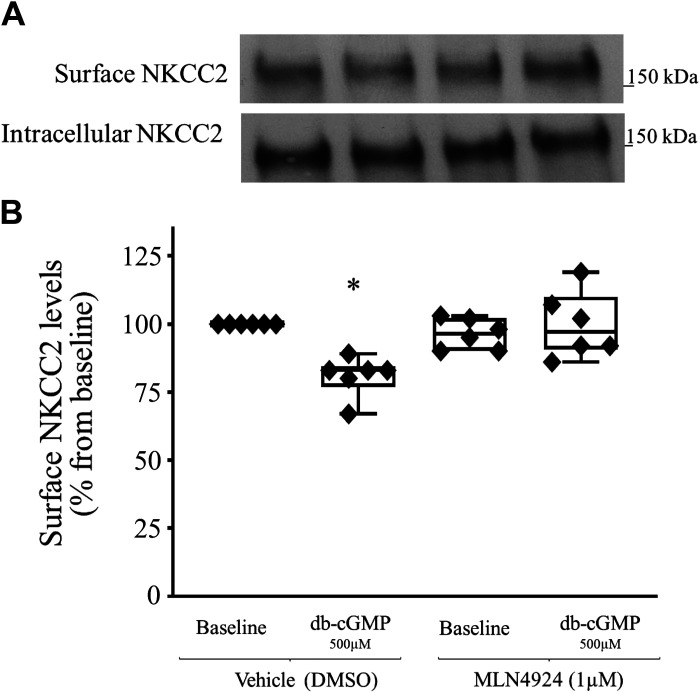
Effect of pharmacological inhibition of cullin activity on surface Na^+^/K^+^/2Cl^−^ cotransporter (NKCC2) levels. Inhibition of cullin neddylation with MLN4924 blunted the cGMP-dependent decrease in surface NKCC2 expression. *A*: representative Western blots showing the effect of dibutyryl cGMP (db-cGMP) on the surface NKCC2 level. A thick ascending limb of loop of Henle (TAL) suspension was equilibrated at 37°C for 10 min with vehicle or a neuronal precursor cell-expressed developmentally downregulated protein 8 activating enzyme (NAE) inhibitor (MLN4924, 1 µM), followed by 20 min of treatment with db-cGMP (500 µM). Intracellular NKCC2 expression did not change with treatment (*bottom*). *B*: cumulative data showing the effect of db-cGMP (500 µM) on surface NKCC2 levels in TALs treated with vehicle or NAE inhibitor (MLN4924, 1 µM; *n* = 5, *P < 0.05 vs. basal). The data (means ± SE) are expressed as the percentage from baseline and were statistically analyzed by one-way ANOVA.

Data are expressed as means ± SE; *n* represents the number of experiments of a particular protocol. A paired *t* test was used to determine significant differences between mean of total protein expression and cGMP-treated samples (see Fig. 7). One-way ANOVA was used to determine significant differences between the mean of surface NKCC2 levels, mean of total NKCC2 levels, and mean ubiquitinated NKCC2 levels, as determined by Western blot analysis, between different treatment groups (see Figs. 1, 2, 4, 6, and 8). A *P* value of <0.05 was considered to indicate statistical significance. Two-way ANOVA with Fligner-Policello adjustment was performed after each Wilcoxon test of two-sample comparisons with unequal variance and a nonnormal data distribution (see Fig. 5).

## RESULTS

### Effect of Pharmacological Inhibition of Cullin Neddylation on NKCC2 Ubiquitination

The second messenger cGMP decreases NaCl reabsorption by decreasing surface NKCC2 levels (4). We have previously shown that ubiquitination and degradation mediate in part the cGMP-dependent decrease in surface NKCC2 levels (7). However, it is unknown which E3 ubiquitin ligases participate in the ubiquitination of NKCC2. We studied whether the inhibition of cullin neddylation would prevent the cGMP-dependent increase in NKCC2 ubiquitination using a compound (MLN4924) that inhibits the binding between Nedd8 and cullins. To test our hypothesis, TALs were suspended in PS containing a proteasomal inhibitor (MG132, 20 µM) to stop the proteasomal degradation of ubiquitinated proteins. TALs were then divided into two groups and treated with either vehicle or MLN4924. Each TAL sample was then divided into two aliquots and treated with saline or db-cGMP (500 µM). We found that inhibition of cullin neddylation blunted the cGMP-dependent increase in NKCC2 ubiquitination (baseline: 100%, db-cGMP: 162.5 ± 8.9%, MLN4924: 75.1 ± 17.5%, MLN4924 + db-cGMP: 96.8 ± 17%, *n* = 5, *P* < 0.05; Fig. 1). The total NKCC2 pool did not differ between the vehicle- and MLN4924-treated samples (baseline: 100.0%, db-cGMP: 99.1 ± 4.1%, MLN4924: 101.1 ± 4.2%, and db-cGMP + MLN4924: 102.3 ± 10.4, *n* = 5, not significant). These data indicate that the cGMP-dependent increase in NKCC2 ubiquitination is mediated by a CRL complex.

### Effect of Pharmacological Inhibition of Cullin Neddylation on Surface NKCC2 Levels in TALs of Sprague–Dawley Rats

We have previously shown that inhibition of proteasomal degradation blunted the cGMP-dependent decrease in surface NKCC2 levels. However, whether preventing the ubiquitination of NKCC2 would blunt the cGMP-dependent decrease in surface NKCC2 levels was unknown. Therefore, we sought to determine whether inhibition of cullin neddylation would prevent the cGMP-dependent decrease in surface NKCC2 levels. First, TALs were divided into two groups and treated with vehicle or MLN4924. TALs were then divided again into two groups and treated with saline or db-cGMP (500 µM). We found that inhibition of cullin neddylation prevented the cGMP-dependent decrease in surface NKCC2 levels (baseline: 100%, db-cGMP: 81.0 ± 3.4%, MLN4924: 96.4 ± 2.2%, and MLN4924 + db-cGMP: 99.8 ± 9.3%, *n* = 6, *P* < 0.05; Fig. 2). These data indicate that inhibition of cullin neddylation blunts the cGMP-dependent decrease in surface NKCC2 levels.

### Expression of Members of the Cullin Family in TALs of Sprague–Dawley Rats

**Figure 3.**
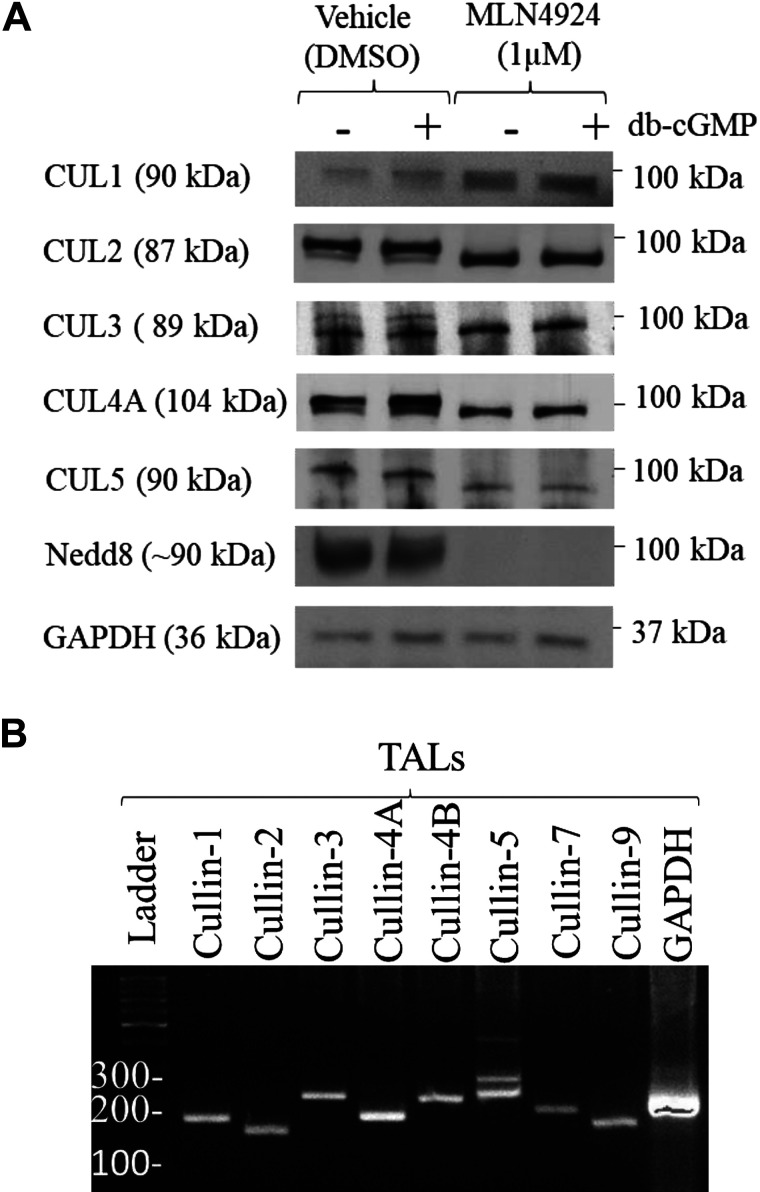
Expression of members of the cullin (CUL) family in thick ascending limbs of the loop of Henle (TALs) from Sprague–Dawley rats. *A*: representative Western blots showing the expression of cullin-1, cullin-2, cullin-3, cullin-4A, and cullin-5 in the TAL of Sprague–Dawley rats (10 µg of protein lysate per lane, *n* = 5). Samples were treated with vehicle or MLN4924 and then divided and treated with dibutyryl cGMP (db-cGMP). Neddylated cullins can be seen at a higher molecular weight in samples treated with vehicle. Cullin neddylation was blunted in samples treated with MLN4924. *B*: reverse transcription was performed using 1 μg of total RNA and 35 cycles of PCR. A 174-bp cDNA for CUL-1, a 154-bp cDNA for CUL-2, a 227-bp cDNA for CUL-3, a 180-bp cDNA for CUL-4A, a 218-bp cDNA for CUL-4B, a 233-bp cDNA for CUL-5, a 194-bp cDNA for CUL-7, a 165-bp cDNA for CUL-9, and a 207-bp cDNA for GAPDH were amplified by PCR. The results shown are representative of experiments using independent mRNA samples from TALs obtained from Sprague–Dawley rats (*n* = 4). Nedd8, neuronal precursor cell-expressed developmentally downregulated protein 8.

To identify the E3 ubiquitin ligases that regulate NKCC2 expression and function in TALs, we examined the expression of members of the cullin family. TALs from Sprague–Dawley rats were treated for 30 min at 37°C with vehicle or MLN4924. We found that cullin-1, cullin-2, cullin-3, cullin-4A, and cullin-5 were expressed in TALs from Sprague–Dawley rats (Fig. 3*A*). We also tested for cullin-4B and cullin-7 expression but were not able to detect these cullins. We found that cullin-1, cullin-2, cullin-3, cullin-4A, and cullin-5 expressed in TALs treated with vehicle migrated as a double band, indicating that the antibody detected both unneddylated and neddylated cullins. As expected, in MLN4924-treated samples, only unneddylated cullins were detected. These data indicate that members of the cullin family are expressed in the TAL and that an inhibitor of cullin neddylation was a suitable tool to test our hypothesis. Since the primary antibodies used have not been fully validated, we studied whether members of the cullin family are expressed in isolated TALs at the mRNA level. To this end, we obtained total RNA from isolated TALs. RNA was converted to cDNA with the reverse transcriptase enzyme. The cDNA was incubated with specific primers (Table 2) specific for each member of the cullin family, and PCR was performed. Our data show that cullin-1, cullin-2, cullin-3, cullin-4A, cullin-4B, cullin-5, cullin-7, and cullin-9 are expressed at the mRNA level in TALs from Sprague-Dawley rats (Fig. 3*B*).

### Effect of Pharmacological Inhibition of Cullin Neddylation on Cullin-1 and Nedd8 Expression in TALs From Sprague–Dawley Rats

**Figure 4.**
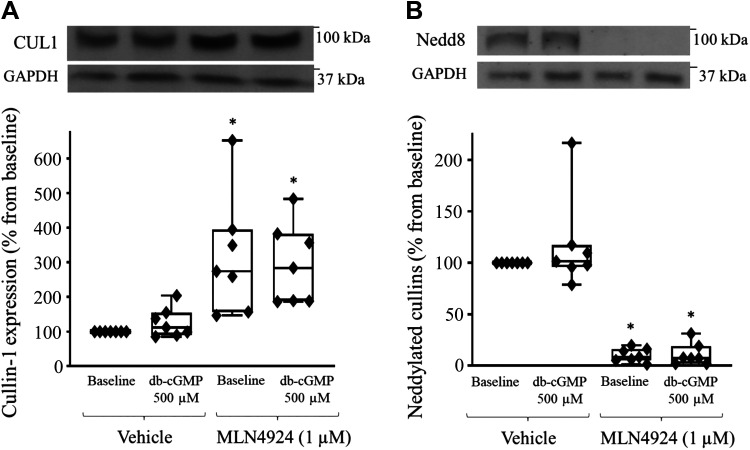
Effect of pharmacological inhibition of cullin (CUL) activity on cullin-1 and neuronal precursor cell-expressed developmentally downregulated protein 8 (Nedd8) expression in the thick ascending limb of the loop of Henle (TAL) of Sprague–Dawley rats. Inhibition of cullin neddylation with MLN4924 increased cullin-1 expression and blunted its neddylation. *A*: cumulative data showing the effect of dibutyryl cGMP (db-cGMP; 500 µM) and Nedd8 activating enzyme (NAE) inhibitor (MLN4924, 1 µg) alone or combined on cullin-1 expression (*n* = 7, *P* < 0.05 vs. basal). *B*: cumulative data showing the effect of db-cGMP (500 µM) in TALs treated with vehicle or NAE inhibitor (MLN4924, 1 µg) on Nedd8 expression (*n* = 7, *P < 0.05 vs. basal). The data (means ± SE) are expressed as the percentage from baseline and were statistically analyzed by one-way ANOVA.

Specific inhibitors for each member of the cullin family that could facilitate their discrimination to determine which mediates the cGMP-dependent increase in NKCC2 ubiquitination are not available. However, we noticed that the expression of only cullin-1 was higher in MLN4924-treated samples. Therefore, we further measured the effect of MLN4924 on cullin-1 expression and cullin neddylation. We found that expression of cullin-1 was increased in MLN4924-treated samples (baseline: 100%, db-cGMP: 130.3 ± 22.5%, MLN4924: 346.0 ± 86.1%, and MLN4924 + db-cGMP: 313.1 ± 58.3%, *n* = 7, *P* < 0.05; Fig. 4*A*). On the other hand, cullin neddylation was blunted in MLN4924-treated samples (baseline: 100%, db-cGMP: 120.3 ± 24.4%, MLN4924: 11.0 ± 3.5%, and MLN4924 + db-cGMP: 11.4 ± 5.8%, *n* = 7, *P* < 0.05; Fig. 4*B*). This finding suggests that cullin-1 is autoregulated by neddylation and is constantly degraded and that, on inhibition of cullin-1 neddylation, cullin-1 expression increases.

### Effect of Pharmacological Inhibition of CAND1 Activity on NKCC2 Ubiquitination in TALs of Sprague–Dawley Rats

**Figure 5.**
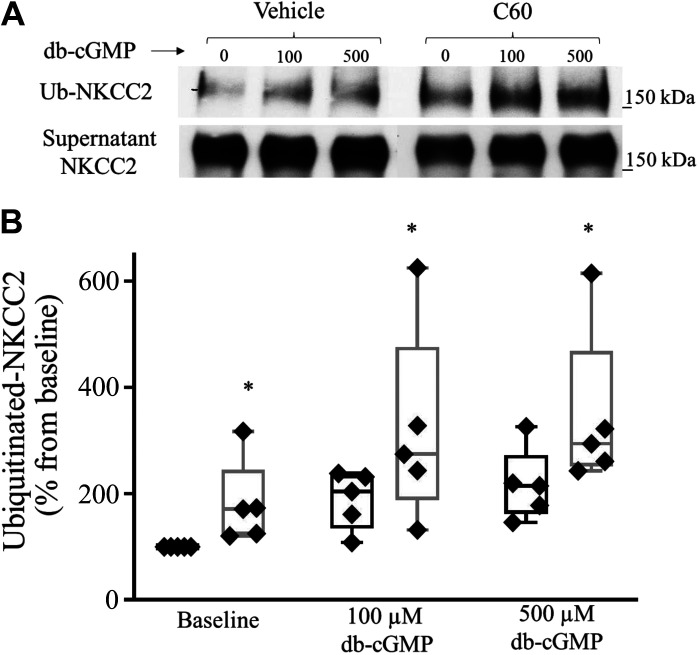
Effect of pharmacological inhibition of cullin-associated neuronal precursor cell-expressed developmentally downregulated protein 8-dissociated 1 (CAND1) activity on Na^+^/K^+^/2Cl^−^ cotransporter (NKCC2) ubiquitination. Inhibition of CAND1 activity with C60 treatment exacerbated the cGMP-dependent increase in NKCC2 ubiquitination. *A*: representative Western blots showing the effect of dibutyryl cGMP (db-cGMP) on NKCC2 ubiquitination. The physiological solution contained a proteasomal inhibitor (MG132, 20 µM) to stop the degradation of ubiquitinated NKCC2. A thick ascending limb of the loop of Henle suspension was equilibrated at 37°C for 10 min with vehicle or a CAND1 inhibitor (C60, 1 µM), followed by 50 min of treatment with 500 µM db-cGMP. Intracellular NKCC2 (*bottom*) expression did not change with treatment. *B*: cumulative data showing the effect of db-cGMP (500 µM) on ubiquitinated NKCC2 in the absence or presence of the CAND1 inhibitor (C60, 1 µM; *n* = 5, *P < 0.05). The data (means ± SE) are expressed as the percentage of the baseline and were statistically analyzed by two-way ANOVA with a Flique–Polciello Wilcoxon test with Hochberg’s correction.

Cullin regulation is dynamic and requires the assembly of several proteins to ubiquitinate a target protein. Although cullin neddylation activates the CRL complex by promoting the assembly of the adaptor protein and the specific substrate-recognition protein, CAND1 prevents the activation of the CRL complex by the displacement of Nedd8, promoting the exchange of substrate-recognition proteins. Therefore, we speculated that inhibition of CAND1 activity would exacerbate the cGMP-dependent increase in NKCC2 ubiquitination. To measure ubiquitinated NKCC2, a proteasomal inhibitor (MG132, 20 µM) was added to the PS. To obtain the required amount of protein for this protocol, TAL suspensions from two Sprague–Dawley rats were combined and mixed. The TAL suspension was then split into two aliquots and treated with either vehicle or 1 µM C60. Later, each TAL sample was further split into three groups and treated with saline, 100 µM db-cGMP, or 500 µM db-cGMP. We found that 1 µM C60 increased NKCC2 ubiquitination as well as the cGMP-dependent increase in NKCC2 ubiquitination (baseline: 100%, 100 µM db-cGMP: 184 ± 31%, 500 µM db-cGMP: 220 ± 39%, C60: 183 ± 46%, C60 + 100 µM db-cGMP: 318 ± 107%, and C60 + 500 µM db-cGMP: 332 ± 96%, *n* = 5, *P* < 0.05; Fig. 5). These data indicate that CAND1 participates in the regulation of NKCC2 ubiquitination under baseline conditions as well as the cGMP-dependent increase in NKCC2 ubiquitination.

### Effect of Pharmacological Inhibition of CAND1 Activity on Surface NKCC2 Levels in TALs of Sprague–Dawley Rats

**Figure 6.**
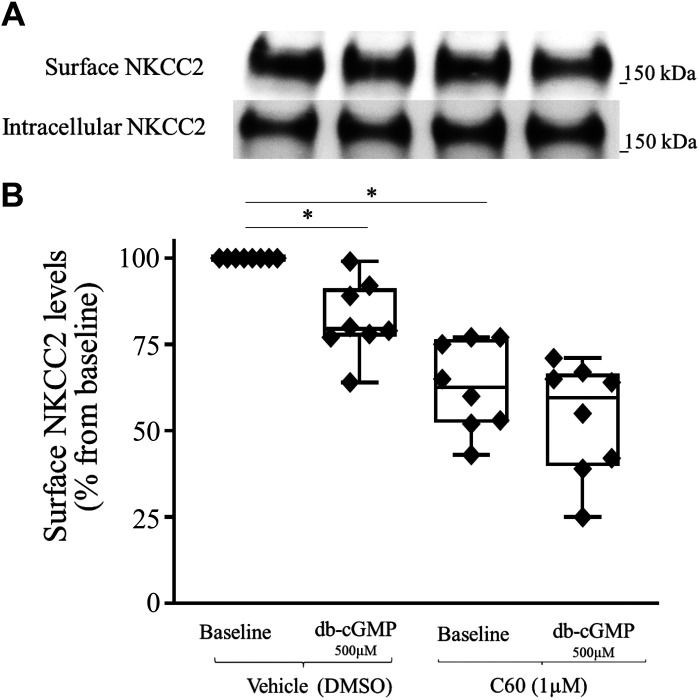
Effect of pharmacological inhibition of cullin-associated neuronal precursor cell-expressed developmentally downregulated protein 8-dissociated 1 (CAND1) activity on surface Na^+^/K^+^/2Cl^−^ cotransporter (NKCC2) levels. Inhibition of CAND1 activity with C60 exacerbated the cGMP-dependent decrease in the surface NKCC2 level. *A*: representative Western blots showing the effect of dibutyryl cGMP (db-cGMP) on the surface NKCC2 level. A thick ascending limb of the loop of Henle suspension was equilibrated at 37°C for 10 min with vehicle or the CAND1 inhibitor (C60, 1 µM), followed by 20 min of treatment with db-cGMP (500 µM). Intracellular NKCC2 (*bottom*) expression did not change with treatment. *B*: cumulative data showing the effect of db-cGMP (500 µM) on the surface NKCC2 level in the absence or presence of the CAND1 inhibitor (C60, 1 µM; *n* = 8, *P < 0.05). The data (means ± SE) are expressed as the percentage from baseline and were statistically analyzed by one-way ANOVA.

Ubiquitination of NKCC2 regulates its plasma membrane expression. Since inhibition of CAND1 exacerbates the cGMP-dependent increase in NKCC2 ubiquitination, we sought to determine whether inhibition of CAND1 would also exacerbate the cGMP-dependent decrease in surface NKCC2 levels. First, TAL samples were divided into two groups and treated with vehicle or 1 µM C60. Each TAL sample was then divided into two groups and treated with saline or 500 µM db-cGMP. We found that inhibition of CAND1 activity exacerbated the cGMP-dependent decrease in surface NKCC2 levels (baseline: 100%, db-cGMP: 82.2 ± 5.9%, C60: 62.8 ± 6.5%, and C60 + db-cGMP: 53.3 ± 8.7%, *n* = 8, *P* < 0.05; Fig. 6). Total NKCC2 expression was not affected by C60 treatment. These data indicate that CAND1 plays a role in the regulation of baseline surface NKCC2 levels as well as the cGMP-dependent decrease in surface NKCC2 levels.

### Effect of cGMP on Nedd8, Cullin-1, and CAND1 Expression in TALs of Sprague–Dawley Rats

**Figure 7.**
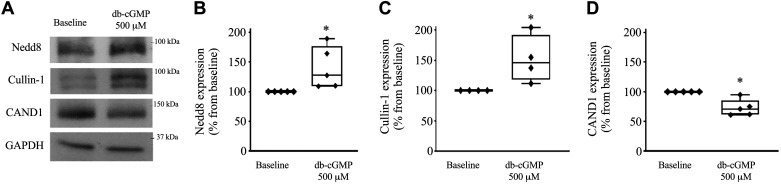
Effect of cGMP on neuronal precursor cell-expressed developmentally downregulated protein 8 (Nedd8), cullin-1, and cullin-associated Nedd8-dissociated 1 (CAND1) expression. *A*: representative Western blots showing expression of Nedd8, cullin-1, CAND1, and GAPDH. *B−D*: cumulative data showing the effect of dibutyryl cGMP (db-cGMP) on Nedd8 (*B*), cullin-1 (*C*), and CAND1 (*D*) expression. A thick ascending limb of the loop of Henle suspension was equilibrated at 37°C for 10 min, followed by 20 min of treatment with 500 µM db-cGMP (*n* = 5, 4, and 4, respectively, *P < 0.05). The data (means ± SE) are expressed as the percentage from baseline and were statistically analyzed by a paired *t* test.

The proteins involved in the mechanism by which cGMP stimulates NKCC2 ubiquitination are unknown. Our data showed that inhibiting the interaction between CAND1 and members of the cullin family exacerbated the cGMP-dependent increase in NKCC2 ubiquitination. Therefore, we speculated that cGMP decreases CAND1 activity or expression. To further assess our hypothesis, we measured the effect of cGMP on Nedd8, cullin-1, and CAND1 expression in TALs by Western blot analysis. TALs were equilibrated for 10 min and then treated with vehicle or 500 µM cGMP for 20 min. We found that cGMP increased Nedd8 expression in TALs (baseline: 100% and db-cGMP: 139.9 ± 15.8%, *n* = 5, *P* < 0.05). cGMP also increased cullin-1 expression in TALs (baseline: 100% and db-cGMP: 151.9 ± 19.5%, *n* = 4, *P* < 0.05). We found that cGMP decreased CAND1 expression in TALs (baseline: 100% and db-cGMP: 75.8 ± 6.9%, *n* = 4, *P* < 0.05; Fig. 7). Our data show that cGMP increases Nedd8 expression while decreasing CAND1 expression, suggesting that cGMP promotes the activation of the CRL complex in TALs.

### Effect of cGMP on Nedd8 Expression in CAND1-Treated TALs From Sprague–Dawley Rats

**Figure 8.**
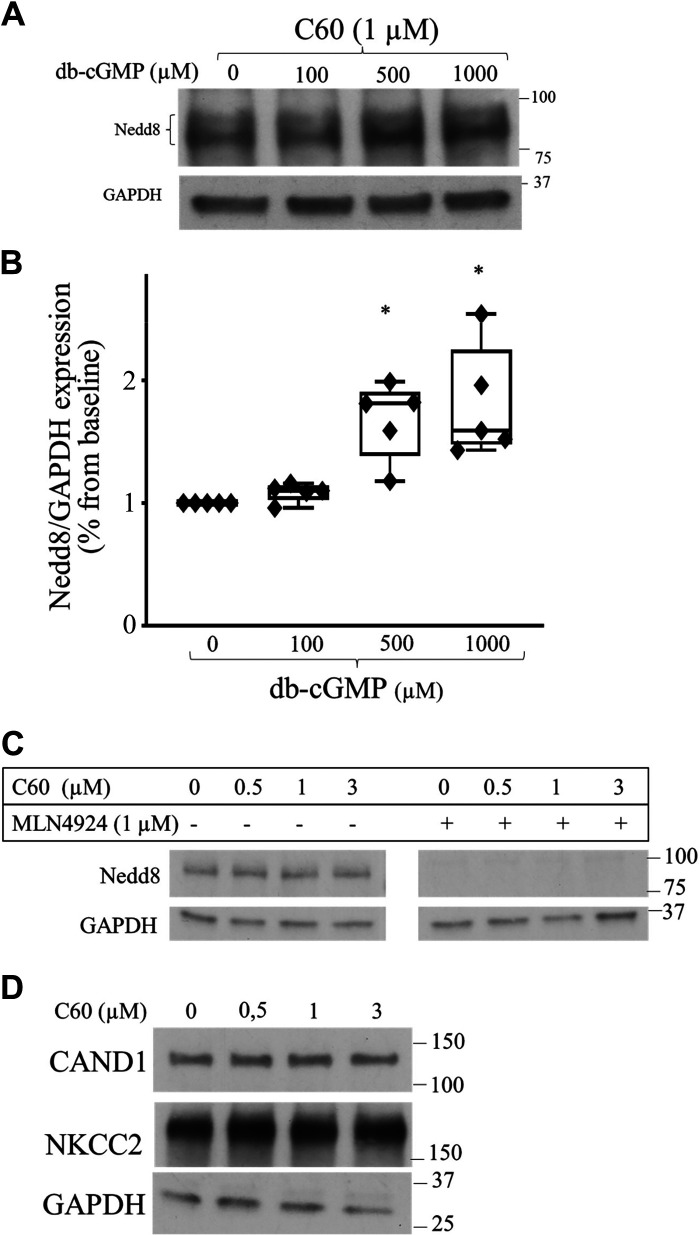
Effect of the pharmacological inhibition of cullin-associated neuronal precursor cell-expressed developmentally downregulated protein 8 (Nedd8)-dissociated 1 (CAND1) activity on cullin neddylation. *A*: representative Western blots showing expression of Nedd8 and GAPDH. *B*: quantitative data showing the effect of cGMP on Nedd8 expression in thick ascending limbs of the loop of Henle (TALs) treated with C60 to inhibit CAND1 activity. TALs were equilibrated at 37°C for 10 min, followed by 50 min of treatment with 100, 500, and 1,000 µM dibutyryl cGMP (db-cGMP; *n* = 6, *P < 0.05). *C*: representative Western blots studying the effect of a concentration-response curve for the novel compound C60 on Nedd8 and GAPDH expression in TALs (*n* = 3, not significant). *D*: representative Western blots studying the effect of a concentration-response curve for the novel compound C60 on CAND1 and total Na^+^/K^+^/2Cl^−^ cotransporter (NKCC2) expression in TALs (*n* = 3, not significant). The data (means ± SE) are expressed as the percentage from baseline and were statistically analyzed by one-way ANOVA.

The mechanism by which cGMP stimulates the CRL complex is unknown. Therefore, we studied whether cGMP promotes neddylation of members of the cullin family in TALs by Western blot analysis. However, since cullins may undergo autoubiquitination and possible degradation, we performed the experiment in the presence of the proteasomal inhibitor MG132 (20 µM) and the CAND1 inhibitor C60 (1 µM). TALs were equilibrated for 10 min and then treated with vehicle or 100, 500, or 1,000 µM cGMP for 50 min. We found that cGMP increased neddylation of members of the cullin family in TALs (baseline: 1.00%, 100 µM db-cGMP: 1.09 ± 0.04%, 500 µM db-cGMP: 1.68 ± 0.16%, and 1,000 µM db-cGMP: 1.81 ± 0.23%, *n* = 5, *P* < 0.05; Fig. 8, *A* and *B*). To ensure that the increase in neddylation is due to cGMP and not inhibition of degradation by the novel compound C60 at the concentration used, the protocol was done in the absence of the proteasomal inhibitor MG132. In the absence of the proteasomal inhibitor MG132, the novel compound C60 did not produce any effect on baseline cullin neddylation (*n* = 3, not significant; Fig. 8*C*). Expression of CAND1 and NKCC2 was not affected by treatment with C60 (*n* = 3, not significant; Fig. 8*D*). Taken together, these data indicate that cGMP stimulates neddylation of members of the cullin family in TALs.

## DISCUSSION

NKCC2 plays an important role in NaCl reabsorption in the TAL (76, 77). In this study, we sought to determine whether NKCC2 ubiquitination is mediated by the CRL complex. We first examined the expression of members of the cullin family. We found that cullin-1, cullin-2, cullin-3, cullin-4A, and cullin-5 were expressed at the protein level, whereas cullin-4B, cullin-7, and cullin-9 were expressed at the mRNA level, in isolated TALs. We found that inhibition of cullin neddylation completely blunted the cGMP-dependent increase in NKCC2 ubiquitination but did not affect baseline NKCC2 ubiquitination. We also found that inhibition of cullin neddylation completely blunted the cGMP-dependent decrease in cell surface NKCC2 levels. In addition, our data showed that inhibition of cullin neddylation increased cullin-1 expression, but this increase in protein expression was not observed for other members of the cullin family. We next studied the role of CAND1 inhibition on NKCC2 ubiquitination and surface expression. Inhibition of CAND1 exacerbated the cGMP-dependent increase in NKCC2 ubiquitination. NKCC2 ubiquitination was increased in CAND1-treated samples, which was accompanied by a decrease in surface NKCC2 levels. In CAND1-treated TALs, the baseline surface NKCC2 level was decreased, and cGMP was not able to further decrease the surface NKCC2 level. Finally, we observed that samples treated with cGMP showed an increase in Nedd8 and cullin-1 expression, accompanied by decreased expression of CAND1. Taken together, these data suggested that cGMP stimulates NKCC2 ubiquitination via a CRL complex. To the best of our knowledge, these are the first data showing that a CRL complex mediates the cGMP-dependent increase in NKCC2 ubiquitination.

Ubiquitination and proteasomal degradation play an important role in the regulation of blood pressure (33) since they regulate NaCl transporters along the nephron (26, 60, 78, 79). We and others have previously shown that NKCC2 is regulated by the ubiquitin-proteasomal system (7, 15–17). However, the specific E3 ubiquitin ligase that mediates NKCC2 ubiquitination remains unknown. We first focused our attention on the largest family of E3 ubiquitin ligases, the CRL complex. The CRL complex binds to substrate-recognition proteins, targeting different proteins for ubiquitination (35). Activation of the CRL complex is initiated by the binding of Nedd8 to a member of the cullin family, a process called neddylation, in which Nedd8 (an 81-amino acid peptide) is covalently attached to a lysine residue in the COOH-terminal portion of a member of the cullin family. NAE, an E1 enzyme, mediates the first step of the reaction by binding Nedd8. This process is followed by the transfer of Nedd8 to an E2-conjugating enzyme. In the final step of the cascade, an E3 ligase enzyme catalyzes the transfer of Nedd8 from E2 to a member of the cullin family. We used the inhibitor MLN4924, which covalently binds NAE (42, 44), to block neddylation of all members of the cullin family. MLN4924 prevented the activation of all CRL complexes.

We have previously shown that inhibition of the proteasomal system regulates surface NKCC2 levels (7). However, whether ubiquitination alone regulates surface NKCC2 levels was unclear. Here, we show that inhibition of NKCC2 ubiquitination (without inhibition of proteasomal degradation) blunted the cGMP-dependent decrease in surface NKCC2 levels. These data indicate that ubiquitination of NKCC2 is required for the cGMP-dependent decrease in surface NKCC2 levels. The data also strongly suggest that a member of the cullin family mediates NKCC2 ubiquitination in TALs.

Our data indicate that the CRL complex mediates the cGMP-dependent decrease in NKCC2 ubiquitination. We examined the expression of members of the cullin family in TALs. We found that in Sprague-Dawley rats, cullin-1, cullin-2, cullin-3, cullin-4A, and cullin-5 were expressed in the TAL. We also tested for cullin-4B and cullin-7 expression, but we were unable to detect these cullins at the protein level. In the present study, we found that inhibition of cullin neddylation blunted the cGMP-dependent increase in NKCC2 ubiquitination. However, inhibition of cullin neddylation did not have a significant effect on baseline NKCC2 ubiquitination or total NKCC2 expression. Interestingly, not all members of the cullin family responded in the same manner. Our data show that cullin-1 expression was three times higher in samples treated with MLN4924, which was not observed for other members of the cullin family. To the best of our knowledge, this is the first report showing an increase in cullin-1 expression in TALs from Sprague–Dawley rats when cullin neddylation is inhibited. However, this was not observed in homogenized whole kidney samples (80). Although the data suggest that cullin-1 is autoregulated, the data do not allow us to conclude that cullin-1 is responsible for mediating the cGMP-dependent increase in NKCC2 ubiquitination. Our data showed that cGMP increased (30−50%) cullin-1 expression (Figs. 3 and 7) when protein content was measured by Western blot analysis. Although we focused on the effect of the second messenger cGMP on cullin-1 due to the changes in expression shown in Figs. 3 and 7, our data do not allow us to determine which member of the cullin family mediates the cullin-dependent increase in NKCC2 ubiquitination.

We also examined expression of each member of the cullin family at the mRNA level. By performing RT-PCR, we found that all members of the cullin family (CUL1, CUL2, CUL3, CUL4A, CUL4B, CUL5, CUL7, and CUL9) were expressed at the mRNA level in TALs. Therefore, expression of cullin-4B, cullin-7, and cullin-9 in the TAL cannot be ruled out based on our Western blot data. Using a proteomic approach, Limbutara et al. (81) dissected tubules from the kidneys of male Sprague–Dawley rats and used protein mass spectrometry, identifying expression of cullin-1, cullin-3, cullin-4A, cullin-4B, and cullin-5, whereas expression of cullin-2, cullin-7, and cullin-9 was at no detectable level. On the other hand, the data from RNA sequencing are currently discrepant. This is based on the data published by Lee et al., who performed a high-throughput method used for global profiling using RNA sequencing coupled with classic renal tubule microdissection from the kidneys of male Sprague–Dawley rats. Deep RNA-sequencing data fail to identify at the mRNA level members of the cullin family in medullary TALs from male Sprague–Dawley rats (82), whereas all members of the cullin family were identified in samples from the mouse kidney (83, 84). Nevertheless, all members of the cullin family have been identified in humans (https://www.proteinatlas.org/search/cullin, https://pdc.cancer.gov/pdc/, and https://www.rebuildingakidney.org/). Taken together, these previous reports align with our findings in which members of the cullin family are expressed at the mRNA and protein level and support our findings that a CRL mediates ubiquitination of NKCC2 in TALs.

However, the role of each member of the cullin family in TAL-dependent NaCl reabsorption remains unknown since the function of each member of the cullin family has not been explored.

Cullins act as scaffold proteins that recruit members of the CRL complex (35). A critical component of the CRL complex is the substrate-recognition protein, which bridges the CRL complex to the protein targeted for ubiquitination. The interaction between a cullin and the substrate-recognition protein is modulated by CAND1 (45, 49). CAND1 was originally reported to selectively bind cullin-1 (45, 47, 48). However, later reports have shown that CAND1 also associates with other members of the cullin family (85, 86). The transient interaction of CAND1 with unneddylated cullin favors the exchange of substrate-recognition proteins, promoting a continuous cycle of substrate adaptor recruitment and displacement (46–48). Therefore, we sought to determine whether inhibition of CAND1 activity would exacerbate the cGMP-dependent increase in NKCC2 ubiquitination. We used the novel compound C60 to inhibit CAND1 activity (53). C60 reduced the association of CAND1 with cullin-1, promoting the association between Nedd8 and cullin and favoring the accumulation of ubiquitinated proteins. Our data show that inhibition of CAND1 increased baseline NKCC2 ubiquitination and exacerbated the cGMP-dependent increase in NKCC2 ubiquitination. These data indicate that CAND1 plays a role in NKCC2 ubiquitination.

The regulation of NKCC2 ubiquitination remains poorly understood. Since inhibition of CAND1 activity increased baseline NKCC2 ubiquitination, these data show, for the first time, that the ubiquitination of NKCC2 can be stimulated. Since NKCC2 ubiquitination regulates surface NKCC2 levels, we examined whether the increase in NKCC2 ubiquitination would be accompanied by exacerbation of the cGMP-dependent decrease in surface NKCC2 levels. Our data show that inhibition of CAND1 with the compound C60 decreased surface NKCC2 levels and that cGMP was not able to further decrease surface NKCC2 levels. To the best of our knowledge, these data show that the ubiquitination of NKCC2 regulates surface NKCC2 levels in TALs.

The regulation of renal transporters is dynamic and involves more than one posttranslational modification. This has been observed in work done with NCC. Rosenbaek et al. (87) showed that stimulation of NCC phosphorylation was accompanied by a decrease in ubiquitination. Moreover, Ishizawa et al. (88) showed that a reduction in K^+^ intake stimulates NCC phosphorylation while decreasing ubiquitination. On the other hand, Murali et al. (80) showed that the increase in NCC ubiquitination due to high K^+^ intake was attenuated when cullin activity was inhibited. Taken together, these studies suggest that an increase in cullin activation is indirectly related to NCC phosphorylation. Furthermore, Murali et al. (80) showed that in the absence of cullin-3, an increase in K^+^ can still reduce phosphorylation of NCC, suggesting that another member of the cullin family may regulate the K^+^-dependent regulation of NCC phosphorylation. Based on the proximity and similarities between NCC and NKCC2, the phosphorylation and ubiquitination of NKCC2 may be regulated in a manner similar to those of NCC. This is based on the fact that although the role of K^+^ on cullin activity in TALs has not yet been studied, Stoke et al. (89) showed that a high-K^+^ diet decreased TAL-mediated NaCl reabsorption, which is in agreement with the recent aforementioned reports. Since NCC and NKCC2 share several conserved motifs, the ubiquitination and phosphorylation of NKCC2 may also be related. However, despite the considerable amount of work done to examine NKCC2 phosphorylation, no study has focused on whether NKCC2 phosphorylation affects the NKCC2 ubiquitination state. Therefore, the interaction between the ubiquitination and phosphorylation of NKCC2 deserves attention.

**Figure 9.**
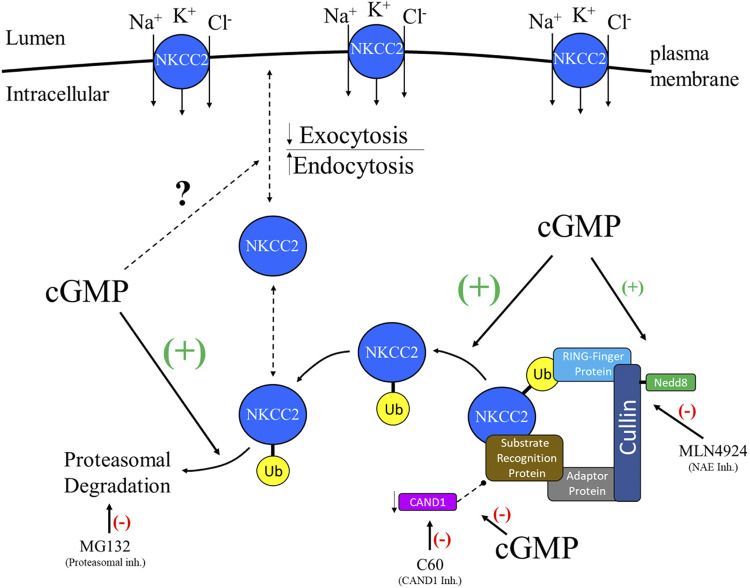
Representative scheme showing the regulation of Na^+^/K^+^/2Cl^−^ cotransporter (NKCC2) by ubiquitination. NKCC2 mediates NaCl reabsorption when is located in the plasma membrane. cGMP decreases surface NKCC2 levels (4, 7). Up to date, it is unknown whether cGMP stimulates endocytosis or inhibits exocytosis (represented by a dashed line). A cullin-RING E3 ubiquitin ligase (CRL) complex (composed of a member of the cullin family, a RING-finger protein, an adaptor protein, and a substrate-recognition protein) is activated by covalent binding between neuronal precursor cell-expressed developmentally downregulated protein 8 (Nedd8) and a member of the cullin family. Once the CRL complex is formed and activated, it binds a target protein (NKCC2) for ubiquitination and subsequent degradation. On the other hand, cullin-associated Nedd8-dissociated 1 (CAND1) promotes the exchange of the substrate-recognition protein, which is essential for the recognition of the targeted protein that undergoes ubiquitination. Therefore, by inhibiting CAND1, the substrate-recognition protein cannot be exchanged, providing stability to the previously assembled CRL complex. Our data indicate that cGMP decreases CAND1 expression in thick ascending limbs of the loop of Henle. Our data suggest that stimulation of cullin neddylation in combination with the decrease in CAND1 expression stimulates formation and activation of the CRL complex. Overall, the data presented throughout this work show that the second messenger cGMP stimulates cullin neddylation, stimulating NKCC2 ubiquitination diverting NKCC2 for proteasomal degradation (7), with a net result of a decrease in surface NKCC2 expression. NAE, Nedd8 activating enzyme.

The data show that cGMP stimulates NKCC2 ubiquitination via a CRL complex. However, the site of action of cGMP is unknown. We found that cGMP increased neddylation of members of the cullin family in rat TALs. We detected an increase in Nedd8 expression, which corresponded to a molecular weight of ∼90–100 kDa, which is the expected molecular weight of cullin family members; since Nedd8 is a small peptide, Nedd8 is detected only with neddylated proteins. Next, we focused on cullin-1, which was expressed at higher levels in cGMP-treated samples. Since the samples were treated with cGMP for only 20 min, we speculate that neddylated cullin-1 was likely protected from degradation and that synthesis of cullin-1 at the protein level was not stimulated. It is also possible that the increase in cullin-1 expression observed may have been due to inhibition of its autoregulation. We studied the effect of cGMP on CAND1 expression. Our data show that cGMP decreased CAND1 expression in native TALs. Currently, little is known about how CAND1 is regulated in mammals. Finally, the data show that the effect of cGMP on neddylation of members of the cullin family was exacerbated when CAND1 was inhibited. Control experiments showed that inhibition of CAND1 did not change baseline neddylation of members of the cullin family nor affect CAND1 expression or total NKCC2 expression. Taken together, the data show that cGMP increases neddylation of members of the cullin family in combination with a decrease in CAND1 expression promoting the activation of a CRL complex, which stimulates ubiquitination of NKCC2. Moreover, the data shown here and in our previous publication demonstrate that the cGMP-dependent increase in NKCC2 ubiquitination leads to the degradation of NKCC2 by the proteasome, decreasing surface NKCC2 levels (7). Up to date, it is not known whether the decrease in surface NKCC2 levels is due to a stimulation of endocytosis, inhibition of exocytosis, or a combination of these trafficking processes (Fig. 9).

Overall, our data show that the cGMP-dependent increase in NKCC2 ubiquitination is mediated by a CRL complex. To the best of our knowledge, this is the first evidence pointing to a specific E3 ubiquitin ligase family that is stimulated by cGMP and has a direct effect on a renal NaCl transporter.

## DATA AVAILABILITY

Data will be made available on reasonable request.

## GRANTS

This work was supported in part by the National Institute of Diabetes and Digestive and Kidney Diseases Grant 1K01DK123192 and Mentored Scientist Institutional support from the Henry Ford Health System.

## DISCLOSURES

No conflicts of interest, financial or otherwise, are declared by the author.

## AUTHOR CONTRIBUTIONS

G.R.A. conceived and designed research; performed experiments; analyzed data; interpreted results of experiments; prepared figures; drafted manuscript; edited and revised manuscript; approved final version of manuscript.
